# *Hericium Erinaceus* Prevents DEHP-Induced Mitochondrial Dysfunction and Apoptosis in PC12 Cells

**DOI:** 10.3390/ijms21062138

**Published:** 2020-03-20

**Authors:** Ines Amara, Maria Scuto, Agata Zappalà, Maria Laura Ontario, Antonio Petralia, Salwa Abid-Essefi, Luigi Maiolino, Anna Signorile, Angela Trovato Salinaro, Vittorio Calabrese

**Affiliations:** 1 Laboratory for Research on Biologically Compatible Compounds, Faculty of Dental Medicine, University of Monastir, Rue Avicenne, Monastir 5019, Tunisia; ines.amara15@yahoo.fr (I.A.); salwaabid@yahoo.fr (S.A.-E.); 2 Department of Biomedical and Biotechnological Sciences, University of Catania, Torre Biologica, Via Santa Sofia n. 97, 95125 Catania, Italy; mary-amir@hotmail.it (M.S.); azappala@unict.it (A.Z.); marialaura.ontario@ontariosrl.it (M.L.O.); calabres@unict.it (V.C.); 3 Department of Medical and Surgery Sciences, University of Catania, 95125, Via Santa Sofia, 78, 95123 Catania, Italy; petralia@unict.it (A.P.); maiolino@policlinico.unict.it (L.M.); 4 Department of Basic Medical Sciences, Neurosciences and Sense Organs, University of Bari, Piazza G. Cesare, 11, 70124 Bari, Italy

**Keywords:** Di (2-Ethylhexyl) pthalate, *Hericium erinaceus*, vitagenes, oxidative stress, apoptosis, mitochondrial respiratory complexes

## Abstract

*Hericium Erinaceus* (HE) is a medicinal plant known to possess anticarcinogenic, antibiotic, and antioxidant activities. It has been shown to have a protective effect against ischemia-injury-induced neuronal cell death in rats. As an extending study, here we examined in pheochromocytoma 12 (PC12) cells, whether HE could exert a protective effect against oxidative stress and apoptosis induced by di(2-ethylhexyl)phthalate (DEHP), a plasticizer known to cause neurotoxicity. We demonstrated that pretreatment with HE significantly attenuated DEHP induced cell death. This protective effect may be attributed to its ability to reduce intracellular reactive oxygen species levels, preserving the activity of respiratory complexes and stabilizing the mitochondrial membrane potential. Additionally, HE pretreatment significantly modulated Nrf2 and Nrf2-dependent vitagenes expression, preventing the increase of pro-apoptotic and the decrease of anti-apoptotic markers. Collectively, our data provide evidence of new preventive nutritional strategy using HE against DEHP-induced apoptosis in PC12 cells.

## 1. Introduction

Phthalates are common plasticizers, used in a large variety of household and medical products to confer flexibility to many polyvinyl chlorides (PVC)–based plastics [1]. Di(2- ethylhexyl)phthalate (DEHP) is one of the most extensively used phthalates, which have a variety of applications including food packages, cosmetics, clothing, children’s toys, and medical devices such as blood storage bags [2]. DEHP is highly hydrophobic compound and it is well absorbed after oral exposure [3], being estimated that human absorption of DEHP could be as high as 25%. Due to its liposolubility, DEHP leaches from plastics following lipophilic fluids [4], making it possible that DEHP crosses the blood-brain barrier into the central nervous system (CNS) tissue, which could lead to neural toxic effects.

Recently, an increasing number of studies have provided evidence of a significant association between DEHP exposure and neuronal disruption. The toxicity of DEHP in mammalian cells has been investigated, and it has been demonstrated that this phthalate is cytotoxic and induces apoptosis in Neuro-2-a cells, a neuroblastome cell line [5]. Other investigators have studied the in vivo administration of DEHP and demonstrated that this plasticizer causes neurodegeneration in the brain of rats [6]. Other lines of evidence indicate that prenatal and postnatal exposure of DEHP affect CNS [7]. Indeed, in utero exposure, DEHP caused disruption of rat brain development [8], while a reduction in the number of mid brain dopaminergic neurons and a motor hyperactivity have been demonstrated after postnatal exposure to this phthalate [9].

An often-mentioned mechanism of chemical-induced neuronal damage is oxidative stress. Excess of reactive oxygen species (ROS), mainly associated with mitochondrial dysfunctions, disrupts antioxidant defense system, generating a vicious cycle resulting in further damage to cellular functions, energy insufficiency, and eventually leading to neuronal cell death [10].

Apoptosis is the most common and well-defined form of programmed cell death which is a genetically directed process of cell self-destruction that is essential for embryonic development, immune-system function, and the maintenance of tissue homeostasis in multi-cellular organisms [11]. It is marked by different changes in the mitochondria such as the release of caspase activators, changes in electron transport, loss of mitochondrial membrane potential (MMP), and involvement of pro and anti-apoptotic Bcl-2 family proteins [12]. In recent years, considerable research has been carried out on identifying naturally occurring substances endowed with neuroprotective properties, impacting on apoptotic processes, and thus capable to prevent or delay neurodegenerative processes. Mushrooms, which have been used in traditional medicine for thousands of years [13,14], have been reported to possess various biological actions, including antitumor, immunomodulatory, antioxidant, antiviral, antibacterial, and hepatoprotective effects [15,16]. Some of the most potent immunostimulatory molecules derived from mushrooms are β glucans, which activate many types of immune cells and stimulate cytokine responses [17,18,19,20]. Administration of complex mixtures of molecules to unknown concentrations is difficult to reconcile with current pharmaceutical practices involving highly purified compounds, and hence, as the active ingredients may be unknown, to patent mushroom extracts is a very difficult task.

Moreover, mushroom-derived polysaccharides are complex molecules that cannot be synthesized, as the mass production of these compounds would require timely and costly extraction processes. As a result, many research efforts have focused on low molecular weight compounds, such as cordycepin, which is a cytotoxic nucleoside analog inhibitor of cell proliferation. Of the mushroom-derived therapeutics, polysaccharopeptides obtained from *Hericium erinaceus* (HE) are commercially the best established. HE is known to have a neuroprotective property evidenced by the regulation of inflammation in relation to the pathology of Alzheimer’s disease. In another modality of protection against Alzheimer’s disease, HE has been shown to stimulate the synthesis of nerve growth factor (NGF) in cultured astrocytes [21]. This growth factor, acting on cholinergic neurons by modulating the activity of two enzymes: cholineacetyltransferase and acetylcholinesterase in Alzheimer’s disease, the activity of these two enzymes is inhibited since the dysfunction of cholinergic neurons is an initial event in Alzheimer’s disease [22].

The ability to sense oxidative and proteotoxic insults and to coordinate defensive stress response are basic elements for cellular adaptation and survival [23,24]. With regard to this, medicinal mushrooms, including HE may have beneficial protective effects in low doses [25,26,27]. Consistently, hormesis dose response is characterized by low dose stimulation and a high dose inhibition. The biphasic dose-response phenomenon is characterized by a U-shaped or inverse dose response curve, depending on the different measured endpoints [28,29,30]. The hormetic dose response results from either a direct stimulation or through an overcompensation stimulatory response following disruption in homeostasis [31]. Such hormetic dose responses provide a quantitative description of the bounds of biological plasticity, and a measure of the extent to which adaptive processes may be upregulated, which is especially relevant to the comprehension of protective effects induced by plant and fungal species [32,33,34,35,36]. It has been known that mushrooms activate the heat shock protein (Hsp) pathway in different brain regions of rats, which plays a crucial role in the cellular stress response [22,37,38], and hence, *Hericium erinaceus biomass* preparations can have neuroprotective effects through modulation of inflammatory processes associated with the neuropathology, as well as through regulation of brain stress response plasticity mechanisms [39]. In addition, antioxidant activity of HE has also been demonstrated in other tissues, including liver [40]. In light of the above-mentioned evidence in the present study, we have investigated in vitro the neuroprotective role of HE biomass preparation against DEHP-induced neurotoxicity.

## 2. Results

**Figure 1:**
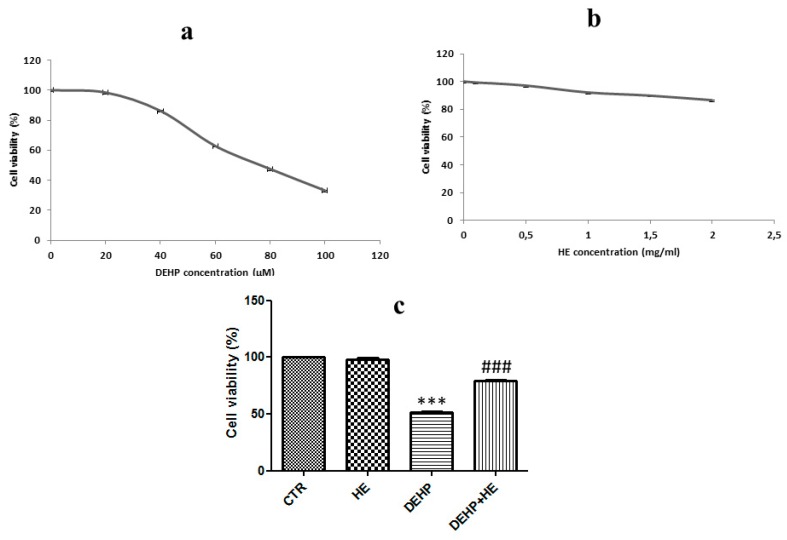
(**a**,**b**) Cytotoxic effect of di(2-ethylhexyl)phthalate (DEHP) and *Hericium Erinaceus* (HE) on pheochromocytoma 12 (PC12) cells. Cells were treated with DEHP or HE at the indicated concentrations for 24 h. (**c**) HE reduces DEHP-induced cytotoxicity in PC12. Cells were pretreated for 2 h with HE (1 µM) before DEHP treatment for 24 h (85 µM). Cell viability was determined using 3-(4,5- Dimethylthiazol-2 yl)-2,5-diphenyltetrazolium bromide (MTT) assay. Data are expressed as the mean ±SD of three separate experiments. *** *p* ≤ 0.001 vs. control and ### *p* ≤ 0.001 vs. DEHP alone.

We first evaluated whether DEHP alone or HE alone treatment was toxic to pheochromocytoma 12 (PC12) cell line. Cells were treated for 24 h with increasing concentrations of DEHP (20, 40, 60, 80, and 100 µM) and HE (0.5, 1 1.5, and 2 mg/mL) (Figure 1a) and cell survival was determined by 3-(4,5-dimethylthiazol-2 yl)-2,5-diphenyltetrazolium bromide (MTT) assay (Figure 1a,b). DEHP treatment induced a dose-dependent reduction in cell viability with approximately IC50 observed at 85 µM (Figure 1a). Consequently, cytotoxic induction with 85 µM DEHP for 24 h was used in the subsequent experiments. Besides, while HE alone exhibited no toxicity towards PC12 cells (Figure 1b), pretreatment with this mushroom at 0.5 mg/mL, significantly decreased DEHP-mediated cytotoxicity (Figure 1c).

**Figure 2:**
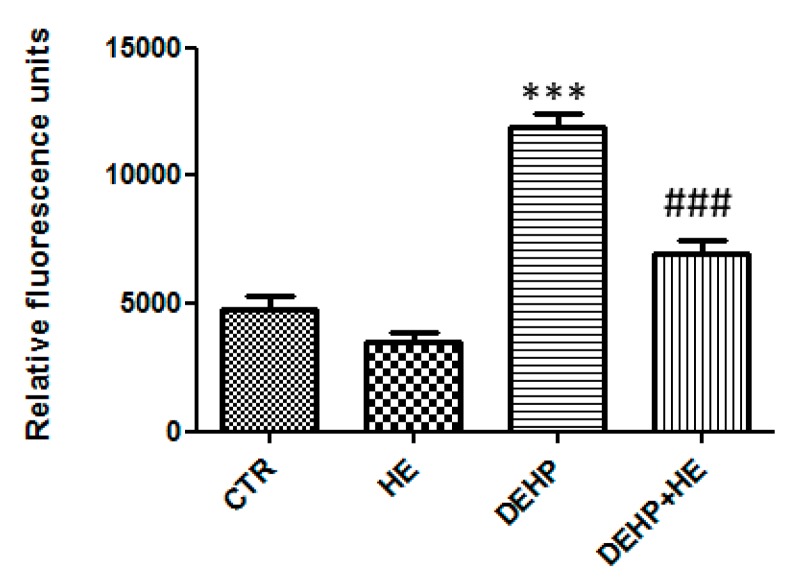
Effects of HE on DEHP-induced reactive oxygen species (ROS) generation. PC12 cells were pretreated with HE (1 µM) for 2 h before DEHP treatment for 24 h (85 µM). The relative intracellular ROS production was evaluated by recording the fluorescence of DCF, the product of 2, 7 - Dichlorofluorescein diacetate (DCFH) oxidation. Data are expressed as the mean ±SD of three separate experiments. *** *p* ≤ 0.001 vs. control and ### *p* ≤ 0.001 vs. DEHP alone.

Indeed, the DEHP treatment results in 51.16 % cell viability, but pretreatment for 2 h with HE at 0.5 mg/mL increase cell viability at 78.73 % (Figure 1c). Thus, we asked whether DEHP exposure affects mitochondrial ROS formation. Therefore, ROS production in PC12 cells was measured using fluorescent dye DCFDA. The levels of intracellular ROS markedly increased after treatment with DEHP. However, pretreatment with HE (0.5 mg/mL) significantly decreased the intracellular ROS generated by DEHP in PC12 cells (Figure 2).

It has been reported that electron transport chain (ETC) complexes are important sources of mitochondrial reactive oxygen species, and their inhibition has been associated with elevated levels of ROS [41]. Given the observed induction of ROS after DEHP treatment, we hypothesized that this excess of ROS may be mediated by mitochondrial dysfunction. To assess this, we analyzed the activity of respiratory chain complexes in PC12 cells treated with DEHP and the effect of HE pretreatment. The data presented in Table 1 shows that the enzymatic activities of complexes I, II-III, IV, as well as ATP synthase drastically decreased after DEHP treatment. However, pretreatment with HE reduces DEHP-induced alterations in mitochondrial respiratory complex activities and significantly restored activity of complex I and II+III (*p* < 0.05).

**Figure 3:**
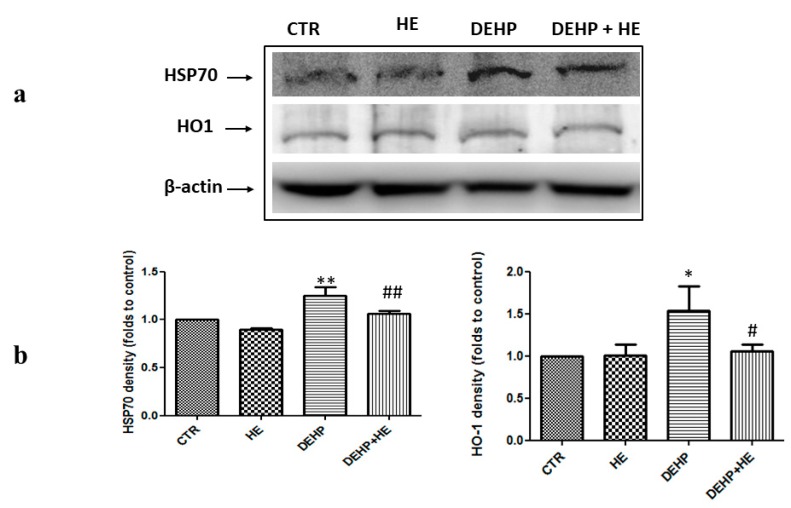
(**a**) The effect of DEHP and HE treatment on HSP70 and HO-1 protein levels. PC12 cells were pretreated with HE (1 µM) for 2 h before DEHP treatment for 24 h (85 µM). The cell lysate proteins were separated by SDS–PAGE, transferred to nitrocellulose membranes, and immunoblotted with the antibodies against HSP70 and HO-1. Protein loading was assessed by re-probing the blots with the β-actin antibody. (**b**) The bars represent the percentage changes of density (folds to control) SD of three independent experiments. * *p* ≤ 0.05 vs. control, ** *p* ≤ 0.01 vs. control, # *p* ≤ 0.05 vs. DEHP alone, ## *p* ≤ 0.01 vs. DEHP alone.

**Figure 4:**
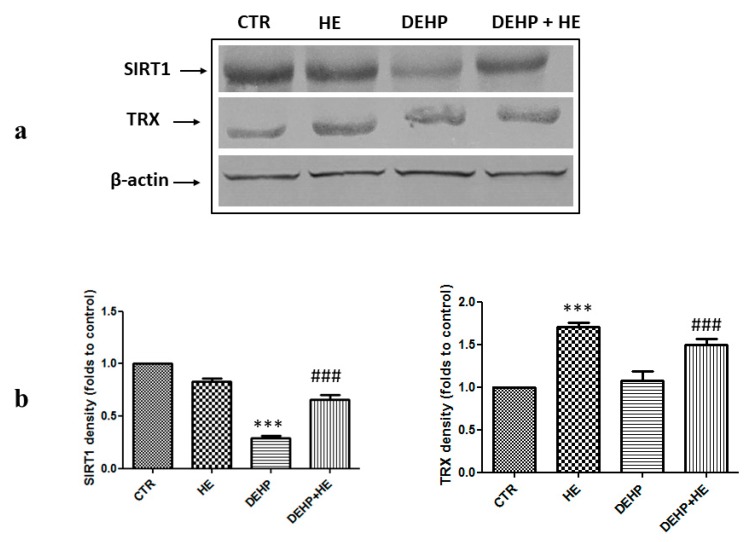
(**a**) The effect of DEHP and HE treatment on SIRT1 and TRX protein levels. PC12 cells were pretreated with HE (1 µM) for 2 h before DEHP treatment for 24 h (85 µM). The cell lysate proteins were separated by SDS–PAGE, transferred to nitrocellulose membranes, and immunoblotted with the antibodies against SIRT1 and TRX. Protein loading was assessed by re-probing the blots with the β-actin antibody. (**b**) The bars represent the percentage changes of density (folds to control) SD of three independent experiments. *** *p* ≤ 0.001 vs. control, ### *p* ≤ 0.001 vs. DEHP alone.

ROS overproduction is accompanied by increased expression of genes participating to recovery of mitochondrial function, detoxification, and cell survival, stress responsive, genes called vitagenes. Vitagenes encode for heat shock proteins (Hsps), thioredoxin, and sirtuin protein systems [42]. The effect of DEHP given alone or combined with HE on vitagenes expression is shown in Figure 3 and Figure 4.

Figure 3a,b and Figure 4b show an increase of HSP70, HO-1, and Trx protein levels induced by DEHP in PC12 cells, whereas this increase was not statistically significant when compared to control for Trx expression. However, DEHP decreased the expression of SIRT1 (Figure 4a). Pretreatment of cells by HE for 2 h, significantly modulates expression of stress responsive vitagenes (Figure 3a,b and Figure 4a,b).

**Figure 5:**
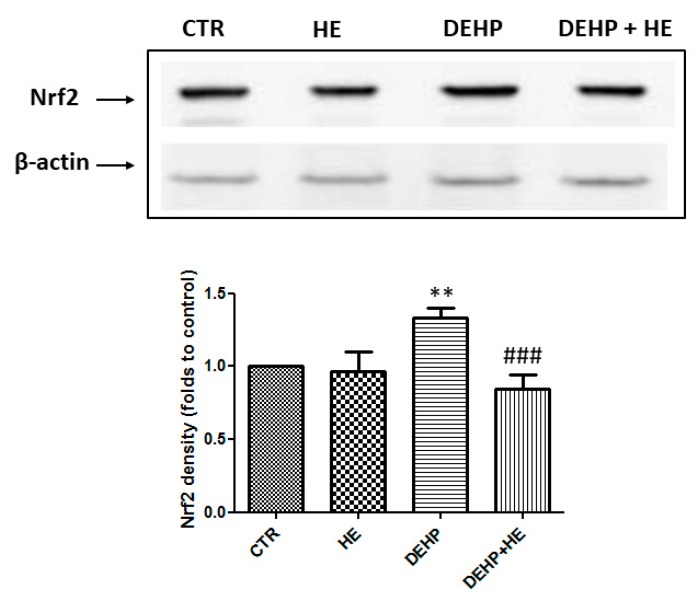
(**a**) The effect of DEHP and HE treatment on Nrf2 protein levels. PC12 cells were pretreated with HE (1 µM) for 2 h before DEHP treatment for 24 h (85 µM). The cell lysate proteins were separated by SDS–PAGE, transferred to nitrocellulose membranes, and immunoblotted with the antibodies against Nrf2. Protein loading was assessed by re-probing the blots with the β-actin antibody. (**b**) The bars represent the percentage changes of density (folds to control) SD of three independent experiments. ** *p* ≤ 0.01 vs. control and ### *p* ≤ 0.001 vs. DEHP alone.

The transcription factor Nrf2 is activated under stressful conditions. Nrf2 binds to the ARE of DNA, leading to the transcription of cytoprotective genes, including HO-1 and Hsp60 [33]. After treatment with DEHP, an increase in the level of Nrf2 was observed, but pretreatment of cells with HE, modulated redox induced Nrf2 expression (Figure 5).

**Figure 6:**
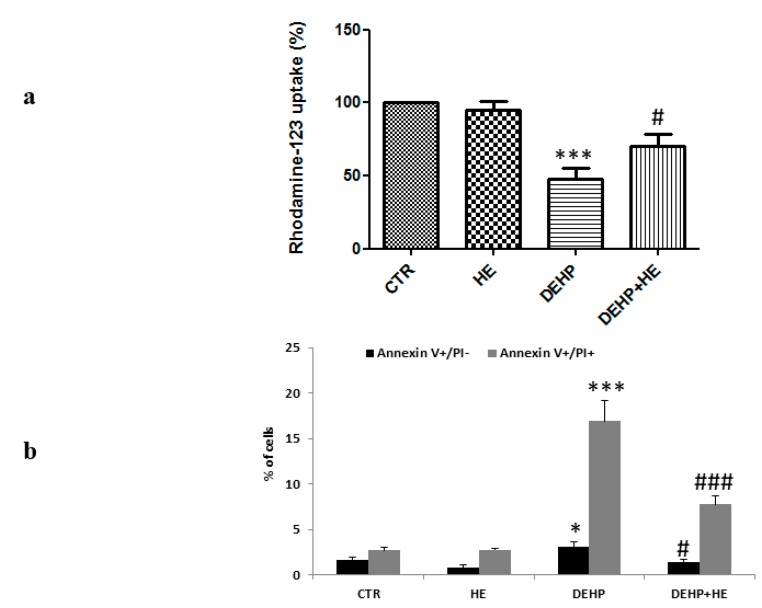
(**a**) Effects of HE on DEHP-induced loss of mitochondrial transmembrane potential. PC12 cells were pretreated with HE (1 µM) for 2 h before DEHP treatment for 24 h (85 µM). The mitochondrial potential was assessed by measuring the uptake of Rhodamine-123. (**b**) Effects of HE on DEHP-induced cell apoptosis. Different subsets of cells were measured by AnnexinV/PI staining after treatment with DEHP (85 µM) and/or HE (1 µM). Early apoptotic cells are positive for AnnexinV and negative for PI (AnnV+/PI-) and late apoptotic/necrotic cells are both positive for AnnexinV and PI (AnnV+/PI+). Data are expressed as the mean ± SD of three separate experiments. * *p* ≤ 0.05 vs. control, *** *p* ≤ 0.001 vs. control, # *p* ≤ 0.05 vs. DEHP alone, ### *p* ≤ 0.001 vs. DEHP alone.

Since mitochondrial anion fluxes are impacted by oxidative modifications, we subsequently examined whether DEHP induced dissipation of the mitochondrial membrane potential (MMP). Rhodamine 123 (Rh123) fluorescence was used to measure the MMP associated with DEHP treatment. As shown in Figure 6a, DEHP treatment reduced the Rh123 uptake to indicating a loss of mitochondrial potential, that was prevented when the cells were pretreated with HE for 2 h.

In addition, double staining cells with FITC-labeled-AnnexinV and PI (Figure 6b) allowed us to confirm by flow cytometry that DEHP induced apoptosis. Compared to the control values, DEHP at 85 µM increased the percentage of early apoptotic cells (AnnV+/PI−) to about 3.1% and late apoptotic/necrotic cells (AnnV+/PI+) to about 16.9% (Figure 6b). Nevertheless, cell pretreatment with HE for 2 h significantly reduced the rate of apoptotic cells (Figure 6b).

Neurodegenerative disorders are associated with mitochondria dysfunctions, alteration of the respiratory chain complexes, MMP decrease, and ROS increase [43]. Excessive ROS generation, MMP disturbance, and modulation of pro and anti-apoptotic proteins play a key role in neuronal apoptosis, thus we tested by western blotting analysis the effect of DEHP on apoptotic protein expression in PC12 cells.

**Figure 7:**
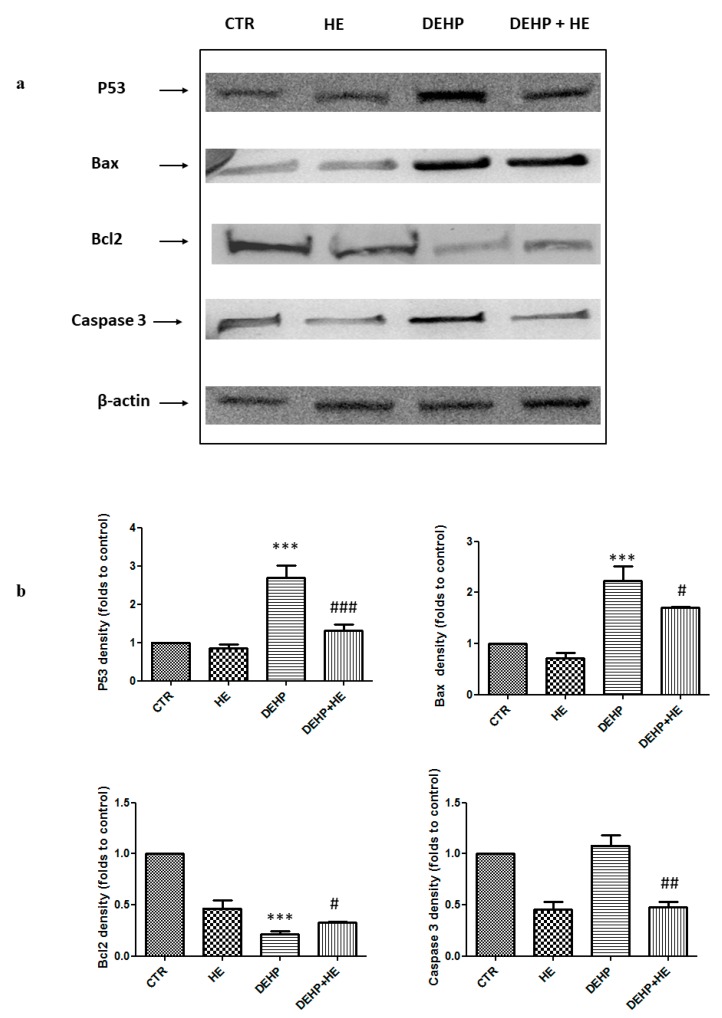

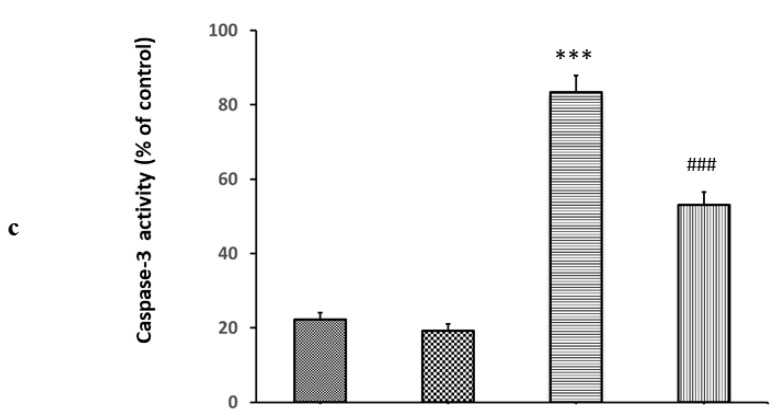
(**a**) The effect of DEHP and HE treatment on P53, Bax, Bcl2, and caspase-3 protein levels. PC12 cells were pretreated with HE (1 µM) for 2 h before DEHP treatment for 24 h (85 µM). The cell lysate proteins were separated by SDS–PAGE, transferred to nitrocellulose membranes and immunoblotted with the antibodies against P53, Bax, Bcl2, and activated caspase-3. Protein loading was assessed by reprobing the blots with the β-actin antibody. (**b**) The bars represent the percentage changes of density (folds to control) SD of three independent experiments. (**c**) The effect of DEHP and HE treatment on caspase-3 activity. PC12 cells were pretreated with HE (1 µM) for 2 h before DEHP treatment for 24 h (85 µM). Caspase-3 activity was measured using a commercialized kit. Data are expressed as the mean ± SD of three separate experiments. *** *p* ≤ 0.001 vs. control, # *p* ≤ 0.05 vs. DEHP alone, ## *p* ≤ 0.01 vs. DEHP alone, ### *p* ≤ 0.001 vs. DEHP alone.

The results in Figure 7 show that the expression of apoptosis biomarker, p53, increased after DEHP treatment (Figure 7a,b). This induction was associated with the overexpression of proapoptotic protein, Bax (Figure 7a,b), and with a decrease in antiapoptotic protein, Bcl2 (Figure 7a,b). All these effects were prevented by pretreatment with HE (Figure 7a,b). We next examined involvement of caspase 3 in apoptosis pathways. Western blotting analysis showed that DEHP treatment resulted in increased level of cleaved active caspase 3 (Figure 7a,b). In addition, caspase-3 activity was measured by fluorimetric assay. As shown in Figure 7c, DEHP treatment significantly increased caspase-3 activity. HE pretreatment was found to be effective to prevent DEHP-induced activation of caspase-3 (Figure 7a–c). Our results indicate the involvement of the caspase-mediated apoptosis pathway.

## 3. Discussion

Although DEHP is extensively used as a plasticizer, few studies have focused on its brain tissue toxicity. In the present study, we investigated the protective effect of HE to counteract oxidative stress, mitochondrial energy deficit and cell death in PC12 cells. Our results showed that HE significantly increased PC12 cells survival against DEHP insult, an effect associated with a decrease in the level of ROS generation, and with modulation of mitochondrial respiratory complex activities, as well as reduction of apoptosis. The present data shows that DEHP induces oxidative stress as demonstrated by robust increase in ROS generation. ROS excessively produced during oxidative stress cause cell damage leading to cell death [33].

Pre-treatment of neuronal cells with HE at the dose of 0.5 mg/mL inhibits intracellular ROS formation and protects cells against DEHP-induced oxidative stress. Protective effects of HE against oxidative stress-induced injuries have been reported in various in vitro and in vivo studies [39,44]. So, we proposed that this anti-oxidative effect of HE may be partly though the possibility of direct elimination of ROS or by rescuing the efficiency of complex I given that the inhibition of this complex is related to ROS overproduction. In fact, it is known that overproduction of ROS might occur through energy dependent mechanisms, mainly a consequence of an inhibition of complex I which generally is associated with cell death [45,46].

To assess the possible alterations in energy metabolism following exposure to DEHP, we measured mitochondrial respiratory complex activities, i.e., NADH-UQ oxidoreductase (complex I), Succinate-cytochrome c oxidoreductase (complex II-III), Cytochrome c oxidase (complex IV) and ATP synthase (complex V). Our results clearly show that DEHP inhibits the mitochondrial respiratory complex I, II-III, IV and V. The mechanism by which DEHP inhibits these complexes is still unclear, however, we hypothesize that inhibition might result from an imbalance in redox status associated with alteration in mitochondrial membrane potential, which can eventually lead to structural disorganization and dysfunction of critical enzymes. Further studies are necessary, however, to determine whether DEHP induces structural and/or post-translational modifications of these enzymes. On the other hand, our results show for the first time that HE treatment could restore the activity of respiratory complexes I, II-III, IV, and V, suggesting that HE may play a direct role in preserving complex activities and eventually prevent ROS production, thus renewing the capacity of neurons to produce energy. Such alterations in respiratory complex activities could be associated with a disturbance of the oxidant/antioxidant balance equilibrium which induces cell degeneration.

Cells are endowed with cellular pathways involved in the maintenance of cellular homeostasis to recovery mitochondrial function and confer protection against oxidative stress [39]. Among these pathways, there is a complex network of the so-called longevity assurance processes, composed of several genes termed vitagenes [27], which include members of the HSP family, such as HO-1, Hsp72, but also sirtuins and the thioredoxin/thioredoxin reductase system [27,42,47]. Molecular chaperones have been known to protect cells against a wide variety of toxic conditions as well as oxidative stress, extreme temperatures or heavy metals exposure. Chaperones play also an important role in the preservation and repair of the correct conformation of the cellular macromolecules, such as proteins, RNAs, and DNA [48]. Indeed, HO-1 catalyzes the degradation of heme and produces carbon monoxide and bilirubin, which can directly scavenge free radicals and repair DNA damage caused by oxidative or nitrosative stresses [39]. Sirtuins are histone deacetylases which, in the presence of NAD^+^ as a cofactor, catalyze the deacetylation reaction of histone substrates and transcriptional regulators. Sirtuins regulate different biological processes, such as apoptosis, cell differentiation, energy transduction, and glucose homeostasis [42]. Furthermore, Trx, is a major redox control system, consisting of a 12 kDa redox active protein Trx, and a homodimeric seleno-protein called thioredoxin reductase (TrxR1). TrxR1 is a flavoprotein that catalyzes the NADPH-dependent reduction of oxidized thioredoxin protein. It is usually located in the cytosol, but it translocates into the nucleus in response to various stimuli associated with oxidative stress, thereby playing a central role in protecting against oxidative stress [27].

In this work we provide experimental evidence that PC12 cells treatment with DEHP for 24 h results in upregulation of vitagenes, in particular Hsp70, HO-1, and Trx and in down regulation of SIRT1. However, our study shows for the first time that HE pretreatment modulates vitagenes expression in PC12 cells. Our results are consistent with evidence obtained in mice, showing neuroprotection by HE on Aβ25–35 peptide-induced cognitive dysfunction [49].

We also provide experimental evidence that upregulation of HO-1 might involve the transcription factor Nrf2, which was highly expressed in the nuclear fraction of cells exposed to DEHP. Nrf2 is a transcription factor that regulates the expression of genes involved in protection against oxidative stress. Nrf2-dependent transcription is under control of the amount of ROS present in cells. Indeed, under basal conditions, Nrf2 is localized in the cytoplasm in its inactive form where it is bound to its inhibitor Keap1 which promotes its degradation by the proteasome via an E3 ubiquitin ligase complex. However, under oxidative stress, Nrf2 dissociates from Keap1, moves into the nucleus, and activates AREs present in promoter regions of a set of genes [50]. Upregulation of Nrf2 observed in our study might be due to oxidative stress generated by this phthalate, which on the other hand, was modulated by HE pretreatment. In addition to this, we found inhibition of the mitochondrial enzyme complexes activities, which can be considered an event preceding the reduction of the mitochondrial membrane potential. Consistent to this finding, excess ROS production by decreasing mitochondrial complex activities and promoting the decline of mitochondrial membrane potential, can induce, as a potent mediator, cell pathway death [39].

We report here that DEHP induces apoptosis through oxidative stress. To understand whether pretreatment with HE can alleviate apoptosis following DEHP exposure, some key factors involved in apoptosis pathway were further evaluated in this study. The apoptotic process is known to be triggered in cells through either the extrinsic or the intrinsic pathway [12]. The intrinsic apoptotic pathway is regulated by the Bcl-2 family proteins which are important modulators of MMP [12]. In order to determine whether Bax, a pro-apoptotic protein, and Bcl2, an anti-apoptotic protein of Bcl-2 family, contributed in the regulation of DEHP-induced decrease of MMP, their expression levels were measured. Our results show that DEHP treatment increased Bax and decreased Bcl2 expressions. Consistent to our findings, it is known that cellular stresses lead to stabilization and activation of the p53 tumor suppressor protein. Moreover, depending on the cellular context, this results in one of two different outcomes: cell cycle arrest or apoptotic cell death. Cell death induced through the p53 pathway is executed by caspase proteinases, which, by cleaving their substrates, lead to the characteristic apoptotic phenotype such as chromatin condensation, plasma membrane asymmetry, and the formation of apoptotic bodies [51]. Our results clearly show that DEHP induces a p53 and caspase-3 activations. These findings confirm that PC12 cells, treated by DEHP, underwent a p53 and caspase-dependent apoptosis. In the other hand, when combined to DEHP (85 µM) and HE (0.5 mg/mL) significantly reduced the apoptosis induced by this phthalate by decreasing the loss of membrane mitochondrial potential, diminishing Bcl2 expression level, increasing Bax, P53, and Caspase-3 expression levels. Although, at the present we cannot precisely indicate whether the apoptotic pathway involved is intrinsic or extrinsic, however our findings support that HE induces an anti-apoptotic activity. It is also known that tryptophan is an essential amino acid and it is a precursor of 5-hydroxytryptamine (serotonin), which is involved in the physiological regulation of several behavioral and neuroendocrine functions [52]. Tryptophan pathway regulated by the rate-limiting enzyme, indoleamine-2,3-dioxygenase (IDO-1), has evolved as a therapeutic target in immunosuppression-induced cancer autoinflammatory diseases [53,54,55,56]. Consistent with this notion, the gut microbiota, which is part of a complex physiological networks, is considered an important source of tryptophan and tryptophan-derived metabolites also involved in neurotransmitter synthesis [57,58,59]. In addition, genetically susceptible individuals with impaired mucosal integrity, undergo escaping of microbial antigens through the epithelial barrier, presenting higher risk for inappropriate immune response and/or underlying chronic inflammation [60]. Our results are relevant to the biology of chronic inflammatory pathology, both in central and peripheral tissues, as many researches have demonstrated that polysaccharides, naturally occurring substances derived from plants or mushrooms [23,37], exhibit favorable therapeutic and health-promoting benefits [61], particularly in relation to diseases associated with inflammation [22,37,38,39]. HE is a medicinal fungus, with the effect of prevention and treatment of gastrointestinal disorders, particularly, owing to its potential therapeutic effect on cancer, promoting immune stimulation and improving lipid metabolism and thus gastrointestinal pathology, where it has been shown that HE supplementation can improve the immune system via regulation of metabolism and composition of gut microbiota [60], thereby reducing ulceration and providing protection against gastric mucosal damage [62]. In conclusion, the present work gives new indication on molecular mechanisms occurring in DEHP exposed PC12 cells, resulting in compensative events against energy defect, and identifies a novel activity of HE to counteract mitochondrial energy deficit, ROS generation, and apoptosis in PC12 cells.

## 4. Materials and Methods

### 4.1. Chemicals

DEHP was purchased from Sigma-Aldrich (St. Louis, MO, USA), *Hericium erinaceus* was from Mycology Research Laboratories Ltd. (Luton, United Kingdom), 3-4,5-Dimethylthiazol-2-yl, 2,5-diphenyltetrazolium bromide (MTT), Cell culture medium Dulbecco’s modified Eagle medium (DMEM), horse serum, fetal bovine serum (FBS), phosphate buffer saline (PBS), trypsin-EDTA, penicillin and streptomycin mixture and l-glutamine (200 mM) were from GIBCO-BCL (UK). 2,7-Dichlorofluoresce diacetate (DCFH-DA) was supplied by Molecular Probes (Cergy Pontoise, France). All other chemicals used were of analytical grade.

### 4.2. Hericium Erinaceus (HE) Biomass Preparation

*Hericium erinaceus* is found almost worldwide; however, its bioactivity varies depending on the habitat in which it grows. To eliminate these variations, established HE-OX strain was used which demonstrates rapid and aggressive colonization. HE, containing both mycelium and primordia (young fruit body) biomass, obtained cultivating the biomass that is grown on a sterilized (autoclaved) substrate. The production process involves the inoculation of sterile organic edible grain with spawn from the mother culture. The fungus is allowed to completely colonize the growth medium aseptically. At the correct stage of development, corresponding to the maximum bioavailability, the living biomass is aseptically air-dried, granulated, tested microbiologically, and reduced in powder for tablet preparation. In comparison to *Hericium* extracts, biomass has the advantage of preserving all nutraceutical potential which is usually reduced with extracts or concentrates, including lyophilisation, and thus the activity of the product corresponds with the source mushroom, while being further intensified by utilizing the entire mycelium. Powder of *Hericium erinaceus* biomass containing mycelium and primordia of the respective mushroom, as the product commercially available, were used for experiments. Optimal concentration was chosen according to previous studies [39,63].

### 4.3. Cell Culture

*PC12* (ATCC^®^ CRL-1721^™^) rat cell line were used for experiments. Cells were cultured in DMEM, supplemented with 10% horse serum, 5% FBS, 1% L glutamine (200 mM), 1% of mixture penicillin (100 IU/mL), and streptomycin (100 µg/mL), at 37 °C in a CO_2_ incubator. Before the experiments, cells were differentiated by culturing in serum-free medium containing 50 ng/mL nerve growth factor (NGF) for 5 days. Protein concentration was estimated using the Bradford assay by spectrophotometrically reading at 595 nm [64].

### 4.4. Cell Viability Assay (MTT)

The cell viability was determined by the MTT assay as described in [65]. This test is based on the ability of living cells to metabolize the yellow tetrazolium salt to a blue formazan via the mitochondrial succinate dehydrogenase which is a member of mitochondrial electron transfer system complex. To determine the neuroprotective effect of HE, PC12 cells (10^5^ cells/well in a 24-well plate) were incubated at 37 °C after pretreatment with HE (1 µM) for 2 h and then incubated with DEHP (83 µM) for 24 h. A negative control containing only cells was also evaluated. After treatment, cells were incubated with 5 mg/mL MTT for 3 h at 37 °C, the medium was removed carefully after the incubation and the formazan crystals were dissolved in 150 μL of DMSO and absorbance of formazan reduction product was measured by spectrophotometer at 570 nm using a microplate reader (Biotek, Elx 800, USA). The results were expressed as the percentage of MTT reduction relative to the absorbance measured from negative control cells. All assays were performed in triplicate. Based on the results obtained from cell viability assay, the effective dose of HE against DEHP toxicity was utilized to study the effect of HE by assessing reactive oxygen species (ROS), mitochondrial membrane potential (MMP), and apoptotic protein marker expression.

### 4.5. Determination of Reactive Oxygen Species (ROS) and Oxidative Stress Status

The intracellular ROS amounts were determined using a fluorometric assay with 2,7-dichlorofluorescein diacetate (DCFHDA). After diffusion inside the cell, the probe is hydrolyzed by intracellular esterase to non-fluorescent dichlorofluorescein (DCFH) and then oxidized to fluorescent DCF in the presence of ROS [66]. PC12 cells were seeded on 24-well culture plates at 10^5^ cells/well for 24 h. After incubation, cells were treated with 20 μM DCFHDA. Intracellular production of ROS was measured after 30 min incubation at 37 °C by fluorometric detection of DCF oxidation on a fluorimeter with an excitation wavelength of 485 nm and emission wavelength of 522 nm. Results are expressed as the ratio of intensity of fluorescence in treated cells to that of the HE responding fluorescence in the control.

### 4.6. Measurement of Mitochondrial Membrane Potential (MMP)

Changes in MMP were determined by the mitochondrial specific, incorporation of a cationic fluorescent dye Rhodamine-123 (Rh-123) [67]. In a typical experiment, the seeded cell in 96- well culture plates were treated with DEHP alone or combined to HE for 24 h. Then cells were rinsed with PBS and 100 μL of Rh-123 (1 μM) in PBS was added on the plates. Cells were incubated (37 °C, 5% CO_2_) for 15 min. Next, the PBS solution containing non-uptaken Rh-123 was washed and replaced by fresh PBS and estimated by fluorimetric detection. The results were expressed as the percentage of uptaken Rh-123 fluorescence relative to the fluorescence measured from negative control cells.

### 4.7. Cell Death Induced by DEHP

To distinguish apoptotic versus necrotic cells, Annexin V/propidium iodide (AnnV/PI) double staining was performed. PI in combination with FITC-AnnV permit to discriminate between viable (AnnV-/PI-), early apoptotic (AnnV+/PI-), and late apopto-tic/necrotic (AnnV+/PI+) cells. The Annexin V assay was performed following the manufacturer’s instructions (Annexin V-FITC kit, Bender MedSystems, Vienna, Austria). Fluorescence of at least 5000 cells was analyzed by flow cytometry.

### 4.8. Preparation of Mitoplasts

PC12 cells were seeded on 6-well culture plates (Polylabo, France) at 1 × 10^5^ cells/well for 24 h of incubation, after, the cells were incubated with DEHP (85 µM) alone or combined to HE (1 µM), for 24 h at 37 °C PC12 cells were collected by trypsinization, pelleted by centrifugation at 500× *g* and resuspended in PBS (pH 7.4). The cell suspension was exposed for 10 min on ice to 2 mg of digitonin/mg cellular proteins. The mitoplast fraction, obtained by digitonin cell disruption, was pelleted at 14,000× *g* and resuspended in PBS [45].

### 4.9. Mitochondrial Respiratory Complex Activity Assay

Mitochondrial complexes activities were determined in mitochondrial membrane-enriched fractions from PC12 cells [45]. Aliquots of trypsinized cells were washed with ice-cold PBS, frozen in liquid nitrogen, and kept at −80 °C until use. To isolate the mitochondrial membrane-enriched fractions, cell pellets were thawed at 2–4 °C, suspended in 1 mL of 10 mM Tris–HCl (pH 7.5), supplemented with 1 mg/mL BSA, and exposed to ultrasound energy for 15 s at 0 °C. The ultrasound-treated cells were centrifuged (10 min at 600× *g*, 4 °C). The supernatant was obtained and centrifuged again (10 min at 14,000× *g*, 4 °C) to collect a mitochondrial pellet that was suspended in 0.1 mL of the respiratory medium. The activity of NADH:ubiquinone oxidoreductase (complex I) was measured in 40 mM potassium phosphate buffer, pH 7.4, 5 mM MgCl_2_, in the presence of 3 mM KCN, 1 μg/mL antimycin, 200 μM decylubiquinone, using 50 μg of mitoplast proteins, by following the oxidation of 100 μM NADH at 340–425 nm (Δε = 6.81 mM^−1^ cm^−1^). The activity was corrected for the residual activity measured in the presence of 1 μg/mL rotenone. [46]. Succinate-cytochrome c oxidoreductase (complex II + III) activity was determined by following its reduction at 550–540 nm in the presence of cytochrome c (Δε = 19.1 mM^−1^ cm^−1^). The activity was also determined in the presence of antimycin A. The activity of Cytochrome c oxidase (complex IV) was established following the ferrocytochrome c oxidation at 550–540 nm (Δε = 19.1 mM^−1^ cm^−1^). Complex V activity (ATP hydrolase activity) was measured by an ATP-regenerating system. Frozen and thawed cells were suspended (at 0.1 mg protein/mL) in a buffer consisting of 375 mM sucrose, 75 mM KCl, 30 mM Tris–HCl pH 7.4, 3 mM MgCl_2_, 2 mM PEP, 55 U/mL lactate dehydrogenase, 40 U/mL pyruvate kinase, 0.3 mM NADH. The reaction was started by the addition of 1 mM ATP and the oxidation of NADH was followed at 340 nm [45].

### 4.10. Protein Extraction and Western Blot Analysis

After treatment with DEHP (85 µM), alone or combined to HE (1 µM), for 24 h at 37 °C, PC12 cells (1 × 10^5^) in 6-well plates were harvested, washed with PBS, and lysed in 100 μL lysis buffer (Hepes 0.5 M containing 0.5% Nonidet-P40, 1 mM PMSF, 1 mg/mL aprotinin, 2 mg/mL leupeptin, pH 7.4), and incubated 20 min in ice before centrifugation. Protein concentrations were determined in cell lysates using Protein BioRad assay. Western blot was carried out as described in [39], proteins extracted for each sample, at equal concentration (50 μg) were boiled for 3 min in sample buffer (containing 40 mM Tris–HCl pH7.4, 2.5 % SDS, 5 % 2-mercaptoethanol, 5 % glycerol, 0.025 mg/mL of bromophenol blue), and then separated on a polyacrylamide mini gels precasting 4-20 % (codNB10420 NuSept Ltd. Australia). Separated proteins were transferred onto nitrocellulose membrane (BIO-RAD, Hercules, CA, USA) in transfer buffer containing (0.05 % SDS, 25 mM Tris, 192 mM glycine, and 20 % v/v methanol). The transfer of the proteins on the nitrocellulose membrane was confirmed by staining with Ponceau Red which was then removed by three washes in PBS (phosphate buffered saline) for 5 min each. Membranes were then incubated for 1 h at room temperature in 20 mM Tris pH 7.4, 150 mM NaCl, and Tween 20 (TBS-T) containing 2 % milk powder and incubated with appropriate primary anti-Hsp70 (SC-10789, Santa Cruz Biotech. Inc), anti-heme oxygenase-1 (HO-1) (SC-10789, Santa Cruz Biotech. Inc), anti-Thioredoxin (Trx) (Sc-13526, Santa Cruz Biotech. Inc.), anti-Sirt1 (SC-74465, Santa Cruz Biotech. Inc.), anti-Bax (SC-7480, Santa Cruz Biotech. Inc, anti-Bcl-2 (SC-7382, Santa Cruz Biotech. Inc), anti-p53 (SC-126, Santa Cruz Biotech. Inc), and anti-caspase-3 polyclonal (Santa Cruz Biotech. Inc.) overnight at 4 °C. The same membrane was incubated with a goat polyclonal antibody anti-beta-actin (SC-1615 Santa Cruz Biotech. Inc., Santa Cruz, CA, USA) to verify that the concentration of protein loaded in the gel was the same in each sample. Excess unbound antibodies were removed by three washes are with TBS-T for 5 min. After incubation with primary antibody, the membranes were washed three times for 5 min. in TBS-T and then incubated for 1 h at room temperature with the secondary polyclonal antibody conjugated with horseradish peroxidase (dilution1:500). The membranes were then washed three times with TBS-T for 5 min. Finally, the membranes were incubated for 3 min with Super Signal chemiluminescence detection system kit (Cod34080 Pierce Chemical Co, Rockford, IL, USA) to display the specific protein bands for each antibody. The immunoreactive bands were quantified by capturing the luminescence signal emitted from the membranes with the Gel Logic 2200 PRO (Bioscience) and analyzed with Molecular Imaging software for the complete analysis of regions of interest for measuring expression ratios.

### 4.11. Caspase-3 Activation Assay

The measure of caspase-3 activity was performed using a commercially available kit, according to the manufacturer’s instructions (BD Pharmingen). At 50% confluence, PC12 cells were cultured in the presence of DEHP alone (85 µM), HE alone (1 µM), or DEHP with HE at 37 °C for 24 h. Cells were harvested and incubated in lysing buffer for 30 min and then incubated with 20 µM Ac-DEVD-AMC (a substrate for caspase-3-like proteases) at 37 °C for 1 h. The release of aminomethylcoumarin (AMC) was then measured on a Perkin-Elmer fluorimeter using an excitation/emission wave length of 380 nm/460 nm. The results were corrected for total protein content in the lysates using the bicinchoninic acid assay (Pierce, Rockford, IL, USA), and expressed as the percentage of activity in lysates from control cultures.

### 4.12. Statistical Analysis

Each experiment was done independently three times. Values are presented as the mean ± standard deviation (SD). The analysis parameters were tested for homogeneity of variance and normality, and they were found to be normally distributed. The data were therefore analyzed using a one-way analysis of variance (ANOVA) with a post hoc Tukey–Kramer test to identify significance between groups and their respective controls. In all cases, *p* < 0.05 (*), *p* < 0.01 (**), *p* < 0.001 (***) was considered statistically significant.

## Figures and Tables

**Table 1:** Effect of HE on di(2-ethylhexyl)phthalate (DEHP) treatment on the activity of Complex I, II–III, and IV and ATP Synthase in PC12 cells.

	NADH-UQ Oxidoreductase Complex I µmol Oxidized NADH/min/mg Protein	Succinate-Cytochrome c Oxidoreductase Complex II-III µmol Reduced Cyt c/min/mg Protein	Cytochrome c Oxidase Complex IV µmol Reduced Cyt c/min/mg Protein	ATP Synthase Complex V µmol Oxidized NADH/min/mg Protein
**CTR**	0.053 ± 0.0036	0.050 ± 0.0013	0.1882 ± 0.0152	0.0066 ± 0.0008
**HE**	0.0452 ± 0.0036	0.043 ± 0.0015	0.1742 ± 0.0078	0.0075 ± 0.00049
**DEHP**	0.0182 ± 0.0007***	0.026 ± 0.0031***	0.0961 ± 0.0173***	0.0034 ± 0.00040**
**DEHP+HE**	0.037 ± 0.0031^#^	0.035 ± 0.0037^#^	0.1218 ± 0.0067	0.0047 ± 0.00032

Effect of HE on DEHP treatment on the activity of Complex I, II–III, and IV and ATP Synthase in PC12 cells. # *p* ≤ 0.05, ** *p* ≤ 0.01, *** *p* ≤ 0.001.
